# Prevalence Rates of Bullying: A Comparison Between a Definition-Based Scale and a Behavior-Based Scale

**DOI:** 10.1177/08862605241262216

**Published:** 2024-07-31

**Authors:** Björn Sjögren, Ylva Bjereld, Robert Thornberg, Jun Sung Hong, Dorothy L. Espelage

**Affiliations:** 1 Linköping University, Sweden; 2 University of Gothenburg, Sweden; 3 Wayne State University, Detroit, MI, USA; 4 University of North Carolina at Chapel Hill, USA

**Keywords:** school bullying, bullying measurement, definition-based, behavioral-based, prevalence

## Abstract

Self-reported measures of school bullying can be divided into two subtypes. Definition-based measures present a bullying definition followed by one question about being bullied and one question about bullying others, while behavior-based measures avoid using terms like “bully” and “bullying,” do not provide an explicit bullying definition, include items describing specific bullying behaviors, and respondents are asked to rate how often they have engaged in or have been a target of each behavior. The current study aimed to compare bullying perpetration and victimization prevalence rates between a definition-based scale and a behavior-based scale. The current study was part of a 4-year longitudinal research project, where students in Sweden completed an annual web-based survey at five waves starting with the school year of 2015 to 2016 (Wave 1; approximately age = 10.5 years) and ending in the school year of 2019 to 2020 (Wave 5; approximately age = 14.5 years). Because they responded to both measurement conditions, the study controlled for their possible individual differences. In this study, data from 1,469 to 1,715 students were analyzed. Findings revealed that the behavior-based scale displayed higher bullying perpetration and victimization prevalence than the definition-based scale. The behavior-based scales used in this study offer researchers and practitioners a self-report bullying measurement that includes power imbalance, concrete, and specific negative behaviors, and the ability to estimate repetition, but without using bullying terminology. Still, pros and cons of both approaches can be further discussed, and both definition-based and behavior-based self-report measures are vulnerable to a number of biases while they provide estimates or approximations—not exact pictures—of bullying prevalence.

## Introduction

Bullying in schools is a global issue that impacts students all over the world (Inchley et al., 2020; Martin et al., 2020). Understanding the prevalence of bullying at individual schools, school districts, nationally, and internationally is critical to advancing the field of bullying prevention. More specifically, researchers and policymakers engage in surveillance of bullying prevalence to track changes in trends over time, to evaluate the efficacy and effectiveness of interventions, and to contribute scientific expertise to larger policy and practice reforms. However, measuring the prevalence of bullying poses certain challenges that have contributed to considerable variation in the prevalence of bullying even within the same cultural context and within the same points in time (Bjereld et al., 2020; Kennedy, 2019; Smith & López-Castro, 2017). For example, a meta-regression on trends of bullying in the United States between 1998 and 2017 identified studies that reported an increase in bullying, studies that reported a decrease in bullying, and those that reported no change at all. These variations were largely dependent on the type of bullying under investigation, such as face-to-face or cyberbullying, as well as on differences across grade and gender (Kennedy, 2019).

### Defining and Measuring Bullying

While many factors may explain differences in the prevalence of bullying across time and context, one of the most important factors is how bullying is defined and measured. Olweus’ (1993) traditional definition of bullying consists of three criteria: an *intention to inflict harm*, *repetition*, and *power imbalance*. While this definition is widely used and cited within the international bullying research community, scholars continue to debate whether this definition and its components accurately capture the current understanding of the nature of bullying. As such, the research literature is replete with definitions that are both similar to Olweus’ definition, but also differ in some respects. For example, Gladden et al. (2014) define bullying as any aggressive unwanted behavior involving “an observed or perceived power imbalance and is repeated multiple times or is highly likely to be repeated . . . and may inflict harm or distress” (p. 7) on the victim. The intention to inflict harm has been removed as a criterion and replaced with *unwanted behavior* that *may cause victim harm/distress*. Volk et al. (2014) also replaced the intention to inflict harm, with a *goal-directed behavior* (e.g., bullying for resources, dominance, and popularity) that *harms* the victim. Certainly, the choice of definition and the measurement of certain components/criteria or exclusion of these criteria inherently contributes to variation in prevalence estimates across studies and over time (Bjereld et al., 2020; Furlong et al., 2010; Volk et al., 2017). Furthermore, bullying can be measured through multiple informants, including teachers, students, and peers; however, the most common procedure to estimate the prevalence of bullying is the use of self-report scales (Thomas et al., 2015; Vivolo-Kantor et al., 2014; Xie et al., 2023). Nonetheless, there exists considerable variation in the types of self-report scales used in the extant research (Vivolo-Kantor et al., 2014; Xie et al., 2023).

### Comparing Definition- and Behavior-Based Scales

Perhaps the most critical decision a researcher must make when designing an investigation on bullying is whether to use the word “bully” in the survey items and whether or not to provide research participants with a definition of bullying prior to the completion of survey items (Vessey et al., 2014). In broad terms, bullying measures can be divided into two subtypes: *definition-based* or *behavior-based* measures (Felix et al., 2011; Furlong et al., 2010; Hunter et al., 2021; Thomas et al., 2015). Definition-based measures are usually constructed as a global question with one single item (e.g., see Shaw et al., 2013; Solberg & Olweus, 2003). This approach involves presenting a definition of bullying followed by one question about the respondents’ personal experiences of bullying others and one question about their experiences of being bullied themselves. In contrast, behavior-based measures often avoid using terms like “bully” and “bullying,” do not provide an explicit bullying definition, and include items that are combined into a bullying index (Demaray et al., 2016; Espelage & Holt, 2001; Martin et al., 2020; Murray et al., 2021). The items describe specific behaviors such as hitting, name-calling, and rumor spreading, and respondents are asked to rate how often they have experienced each behavior in a specified timeframe (Furlong et al., 2010).

Studies using behavior-based measures typically report higher prevalence rates of bullying than do those studies using definition-based measures (Beltran-Catalan & Cruz-Catalan, 2020; Bjereld et al., 2020; Hellström et al., 2013; Huang & Cornell, 2015; Xie et al., 2023). There are some plausible explanations for these differences. First, it is well-established in the research literature that bullying is often associated with shame, guilt, and stigma for both students who are bullying others and those that are being bullied (Bjereld, 2018; Jia & Mikami, 2018; Xie et al., 2023). Thus, using definitions or items that explicitly mention bullying might provoke strong negative emotions, like guilt or shame, and in turn, may contribute to underreporting (Felix et al., 2011; Furlong et al., 2010; Jia & Mikami, 2018; Thomas et al., 2015). Second, oftentimes these definitions can become lengthy and therefore require considerable time, attention, and energy for participants to read and fully comprehend. For this reason, some scholars have questioned whether students are reading the definition in its entirety and considering all components (e.g., intentionality and power) when responding to the follow-up items (Huang & Cornell, 2015; Ybarra et al., 2012). Third, others have argued that behavior-based scales that do not provide a definition are likely tapping into general peer aggression and victimization in addition to bullying (Bjereld et al., 2020), thereby failing to adhere to the definitional criteria of bullying (Furlong et al., 2010). One way to counteract this risk of capturing general peer aggression is to assess directly the components of bullying (e.g., power imbalance, repetition) without using a definition or words like “bully” or “bullying” (Felix et al., 2011; Varjas et al., 2009).

Some studies employing definition-based measures present the definition followed by two global questions, one that assesses perpetration and one that assesses victimization, while other studies employ a hybrid approach where the definition is followed by several questions that query the frequency of specific forms of bullying, including verbal, physical, social (Huang & Cornell, 2015; Vaillancourt et al., 2010). Prevalence rates tend to vary depending on the order of the questions. For example, Vaillancourt et al. (2010) found that when students responded to one global question of bullying, 38% reported that they had been bullied, but when they responded to the subsequent list of items, 63% reported that they had been a target of at least one of the more specific behaviors. Further, an experimental study by Huang and Cornell (2015) showed that when students were asked questions on specific forms of bullying behavior before one global question, victimization prevalence rates increased 29% to 76% on the specific items. These authors conclude that surveys where specific types of bully victimization experiences are assessed after a general, global question may underestimate prevalence rates, and suggest that specific items are assessed prior to specific questions on bullying (Huang & Cornell, 2015).

Although the bullying research field has grown tremendously during recent decades, there continues to be little consensus among scholars on the best approach to measuring the prevalence rates of bullying. This lack of consensus is partly a result of few comparative studies examining definition- and behavior-based measures in the same study. Without experimental studies that compare and contrast these approaches, interpreting differences in prevalence rates is hampered by other confounding factors, such as study populations, age groups, and other methodological approaches.

### The Current Study

The current study aimed to compare bullying perpetration and victimization prevalence rates between a definition-based scale and a behavior-based scale among fourth- to eighth-grade students who completed both measures. Differences in prevalence rates across the two measures were examined for entire sample, but also were examined by gender.

## Method

### Participants

The current study was part of a 4-year longitudinal research project in Sweden, where a cohort of students completed an annual web-based survey across five waves, starting in fourth grade during the 2015/2016 school year (Wave 1) and ending in eighth grade during the 2019/2020 school year (Wave 5). In the Swedish school system, the compulsory school consists of kindergarten/preschool class (the year children turn 6 years old), lower elementary school (grades 1–3), upper elementary school (grades 4–6), and lower secondary school (grades 7–9). In elementary school, students have one classroom (homeroom) in which most of their classes occur, and they have the same classroom teacher for many or most subjects. In lower secondary school, students have a variety of subject teachers, and they change classrooms for each subject.

In the first wave, 2,408 fourth-grade students were invited to participate. Out of these, 599 students did not acquire parental consent, and 183 students were absent on the day of data collection or chose not to participate. Furthermore, 87 students did not fill out all the scales used in the study and were therefore excluded. Thus, Wave 1 consisted of data from 1,539 students (mean age = 10.54, *SD* = 0.35, 52% girls). In each of the following data collection waves, some students chose to withdraw from the study, were absent on the day of data collection, or had transferred to schools not involved in the project, whereas some students joined in. In fifth to eighth grade (Waves 2–5), data were obtained from 1,469 to 1,715 students (see Table 1 for descriptive statistics of the participants).

**Table 1.** Descriptive Statistics of Participants.

Time Point	*N*	*M*_age_	*SD*_age_	% Girls
Timepoint 1/grade 4	1,539	10.54	0.35	53
Timepoint 2/grade 5	1,638	11.55	0.33	52
Timepoint 3/grade 6	1,469	12.58	0.35	53
Timepoint 4/grade 7	1,715	13.65	0.36	53
Timepoint 5/grade 8	1,574	14.57	0.34	53

Socioeconomic data were not gathered on an individual level. However, the sampling procedure of selecting schools was strategic in that our sample was diverse and included students from socioeconomically and socio-geographically diverse locations in Sweden (from lower-class to upper-middle-class; rural areas, small towns, medium-sized, and large cities). These schools were situated in 10 municipalities across central and southern Sweden, encompassing a wide range of population sizes, from around 10,000 to 160,000 residents. Specifically, five municipalities fell within the 10,000 to 50,000 population range, two had populations between 50,000 and 100,000, and three had over 100,000 inhabitants. One of the municipalities was part of a metropolitan area. Over the course of five data collection waves, the composition of participants included 19% to 21% with an immigrant background, defined as either not being born in Sweden or having two foreign-born parents. This compares to the national average of 23% to 26% during the corresponding school years (Swedish National Agency for Education, 2023). On average, 53% to 55% of students attending these schools had at least one parent who had pursued post-secondary education, which closely mirrors the broader Swedish school system where this figure stood at 56% to 58% between the school years 2015/2016 and 2019/2020 (Swedish National Agency for Education, 2023). Notably, there was substantial variability among schools in terms of the educational attainment of students’ parents, with the proportion of students having at least one parent with post-secondary education ranging from 22% to 91%, thus highlighting the diversity within the sample.

### Procedure

Before conducting the study, ethical approval was obtained from the Regional Ethical of Linköping. School principals and teachers were informed about the research, and the researchers were given permission to discuss the project with the students. Both written informed parental consent and student assent were obtained from all participants. The students who took part in the study completed a web-based survey on tablets while at school. Before filling out the survey, participants were given standardized instructions and were assured that their participation was both confidential and voluntary and could withdraw from the study at any time without the need to provide a reason. On average, it took 20 to 30 min to complete the survey.

### Measures

#### Behavior-Based Scales

The behavior-based scale for measuring bullying victimization and perpetration, respectively, took the form of two 11-item self-report scales: the School Bullying Perpetration Scale (SBPS) and the School Bullying Victimization Scale (SBVS) (see Supplemental Appendix A). Participants were not provided with a definition of bullying and the survey stem and items did not include the word “bully.” Power imbalance and repetition were still measured on the scales. To assess perpetration, students were presented with the following stem, “Think of the past three months in school. How often have you done the following things toward someone who is weaker, less popular, or less powerful in comparison to you?” To assess victimization, students were asked “Think of the past three months in school: How often have one or more students who are stronger, more popular, or more powerful in comparison to you done the following things to you?” The 11-item scales included five physical (e.g., “Pushed the students so that it hurt, or so that he/she fell down” [perpetration specific]/“Pushed me hard so that it hurt or that I fell down” [victimization specific], three verbal (e.g., “Teased the students and called him/her mean names” [perpetration specific]/“Teased me or called me mean names in a way that bothered me” [victimization specific]), and 3 relational (e.g., “Excluded him/her from our group” [perpetration specific]/“Excluded me from their group” [victimization specific]) forms of bullying. Response options were on 5-point scale: *I haven’t done this* (perpetration specific)/*has not happened to me* (victimization specific) (1), *only a few times* (2), *2 or 3 times a month* (3), *about once a week* (4), and *several times a week* (5). The internal consistency was good within our samples as indicated by Cronbach’s αs ranging from .85 to .93 for bullying perpetration and from .88 to .92 for bullying victimization. Five separate confirmatory factor analyses, one for each wave, were run for SBVS and SBPS, respectively, and provided support for the one-dimensionality of the scales (see Table 2). The confirmatory factor analyses were estimated using unweighted least squares with adjusted mean and variance, which uses the estimated polychoric correlation matrix and is appropriate for ordered categorical data (Klauth, 2023). The mean scores of the victimization and perpetration items at each data collection wave were computed as index variables for bullying victimization and bullying perpetration, respectively. The behavior-based questionnaire on victimization was the sixth questionnaire of the survey, and the behavior-based questionnaire on perpetration was the eighth questionnaire of the survey.

**Table 2.** Fit Indices for CFA Models of SBVS and SBPS.

CFA	χ^2^	*df*	CFI	RMSEA [90% CI]	SRMR
SBVS					
Timepoint 1/grade 4	276	44	0.988	0.038 [0.03, 0.04]	0.049
Timepoint 2/grade 5	563	44	0.981	0.058 [0.05, 0.06]	0.067
Timepoint 3/grade 6	587	44	0.976	0.057 [0.05, 0.06]	0.078
Timepoint 4/grade 7	729	44	0.958	0.074 [0.07, 0.08]	0.096
Timepoint 5/grade 8	742	44	0.975	0.073 [0.07, 0.08]	0.084
SBPS					
Timepoint 1/grade 4	133	44	0.987	0.010 [0.01, 0.01]	0.055
Timepoint 2/grade 5	191	44	0.974	0.015 [0.01, 0.02]	0.065
Timepoint 3/grade 6	181	44	0.974	0.013 [0.01, 0.02]	0.069
Timepoint 4/grade 7	278	44	0.983	0.023 [0.02, 0.03]	0.062
Timepoint 5/grade 8	200	44	0.991	0.022 [0.02, 0.03]	0.048

*Note*. CFA = confirmatory factor analysis; CFI = comparative fit index; RMSEA = root mean square of error of approximation; SRMR = standardized root mean square residual; SBVS = School Bullying Victimization Scale; SBPS = School Bullying Perpetration Scale.

#### Definition-Based Scales

For measuring bullying perpetration and victimization using a definition-based approach, we used the Swedish version (Olweus, 1996a) of the Revised Olweus Bully/Victim Questionnaire (Olweus, 1996b). The scale starts by providing the following definition of bullying:

Here are some questions about being bullied by other students. First, we explain what bullying is. We say a student is being bullied when another student, or several other students:

- say mean and hurtful things, or make fun of him or her, or call him or her mean and hurtful names;
- completely ignore or exclude him or her from their group of friends or leave him or her out of things on purpose;
- hit, kick, push, shove around, or lock him or her inside a room;
- tell lies or spread false rumors about him or her or send mean notes and try to make other students dislike him or her;
- and do other hurtful things like that.

When we talk about bullying, these things happen more than just once, and it is difficult for the student being bullied to defend himself or herself. We also call it bullying, when a student is teased more than just once in a mean and hurtful way.

But we don’t call it bullying when the teasing is done in a friendly and playful way. Also, it is not bullying when two students of about equal strength or power argue or fight.

Once the students read the definition, they were presented with two questions: one about bullying perpetration (i.e., “How often have you taken part in bullying another student(s) at school in the past couple of months?”) and one about bullying victimization (i.e., “How often have you been bullied in school in the past couple of months?”). Response options were on a 5-point scale: *I haven’t bullied* (perpetration specific)*/I haven’t been bullied* (victimization specific) (1), *once or twice* (2), *2 or 3 times a month* (3), *about once every week* (4), and (5) *several times a week*. The definition-based questionnaire was placed at the end of the survey.

### Statistical Analyses

McNemar tests were conducted to investigate whether the bully and victim prevalence rates differed significantly between the definition-based (DB) and the behavior-based (BB) scales. McNemar is a non-parametric test suitable for analyzing paired nominal data (Smith & Ruxton, 2020). Two cutoff scores were examined for the definition-based measure: (a) 2 to 3 times a month; (b) once a week. Four different cutoff values were examined for perpetration and victimization for the behavior-based measure: (a) prevalence rates of bullying at least 2 to 3 times a month on at least one behavior item; (b) prevalence rates of bullying at least 2 to 3 times a month on at least two behavior items; (c) prevalence rates of bullying once a week on at least one behavior item; and (d) prevalence rates of once a week on at least two behavior items. For each grade, four tests were conducted for perpetration, and four tests were conducted for victimization (for a total of 20 tests for perpetration and 20 tests for victimization): (a) comparison between DB and BB (cutoff = 1 item; cutoff = 2–3 times/month); (b) comparison between DB and BB (cutoff = 2 items; 2–3 times/month); (c) comparison between DB and BB (cutoff = 1 item; cutoff = once a week); and (d) comparison between DB and BB (cutoff = 2 items; cutoff = once a week). Odds ratios were calculated to estimate effect sizes. Regarding the choice of cutoff values for repetition, it is important to note that there are no universal levels. Being involved in bullying 2 to 3 times a month has been suggested as a useful lower-bound cutoff point that clearly distinguishes those involved in bullying from others, and is associated with more negative outcomes (Solberg & Olweus, 2003; van der Ploeg et al., 2015; Ybarra et al., 2014). However, different studies use different cutoff values, and it is likely that being involved in bullying even more frequently is associated with even worse outcomes (Malecki et al., 2015). Therefore, in this study, we chose to include both 2 to 3 times a month and once a week as cutoff criteria.

## Results

### Descriptive Statistics

Table 3 presents percentages of students involved in bullying perpetration and bullying victimization according to the definition-based and the behavior-based scale and according to different cutoff criteria. The levels of bullying perpetration increased over time, irrespective of which scale and cutoff criteria were used. Overall, there were higher percentages for victimization compared to perpetration. Across grades, 0.6% to 4% of the students were involved in perpetration according to the definition-based scale, whereas 1.2% to 17% of the students were involved in perpetration according to the behavior-based scale. The prevalence of bullying perpetration was 3 to 8 times higher for the behavior-based scale when using one item as the cutoff criterion and 1.2 to 3.5 times higher when using two items as the cutoff criterion. For victimization, 3.5% to 10% and 11% to 40% of the students were involved in victimization according to the definition-based and the behavior-based scale, respectively. The prevalence of bullying victimization was 3.5 to 6 times higher for the behavior-based scale when using one item as the cutoff criterion and 2.1 to 3.7 times higher when using two items as the cutoff criterion.

**Table 3.** Percentages of Students Involved in Bullying Perpetration and Bullying Victimization According to the Definition-Based and the Behavioral-Based Scale.

Measure	Cutoff Criteria		G4 (%)	G5 (%)	G6 (%)	G7 (%)	G8 (%)
	*n* Items	Frequency					
Bullying perpetration							
Behavior-based	1	2–3 Times/month	7.5	8.9	10.9	14.4	17
Behavior-based	2	2–3 Times/month	3.9	4.5	4.6	7.3	10
Definition-based	—	2–3 Times/month	1.8	1.8	1.4	2.6	4
Behavior-based	1	Once a week	2.9	3.5	3.0	6.9	8
Behavior-based	2	Once a week	1.2	1.6	1.2	3.8	5
Definition-based	—	Once a week	1.0	0.9	0.6	1.1	2
Bullying victimization							
Behavior-based	1	2–3 Times/month	38.7	39.8	39.3	39.8	36.1
Behavior-based	2	2–3 Times/month	24.7	26.4	25.5	25.9	24.5
Definition-based	—	2–3 Times/month	9.7	10	9.7	7.6	7.4
Behavior-based	1	Once a week	20.4	22.8	19.3	22.7	21.3
Behavior-based	2	Once a week	11.4	13.6	11.8	13.5	12.8
Definition-based	—	Once a week	5.3	5.3	4.7	3.8	3.5

*Note*. G4 = grade 4; G5 = grade 5; G6 = grade 6; G7 = grade 7; G8 = grade 8.

### Comparison of the Definition-Based and Behavior-Based Scale

To examine whether bullying perpetration and victimization prevalence rates of the definition-based scale and the behavior-based scale differed significantly from each other, we carried out McNemar tests. In most cases, McNemar tests revealed that the behavior-based scale displayed significantly higher bullying perpetration prevalence than the definition-based scale (for an overview, see Table 4). There were, however, no differences between the definition-based and behavior-based scales in fourth and sixth grades when using once-a-week and two items as cutoff criteria. For bullying victimization, the behavior-based scale displayed significantly higher prevalence in all cases (for an overview, see Table 5). Furthermore, among all the significant differences for bullying perpetration and victimization, all but one were highly significant (<.001). Odds ratios of the significant effects ranged from 2.9 to 36 for bullying perpetration and from 4.9 to 65.7 for bullying victimization. Finally, to investigate potential gender differences, we also ran separate McNemar tests for boys and girls and found similar results for both groups. The only difference was that for girls, there was no difference between the definition-based and behavior-based scale in fifth grade when using once-a-week and two items as cutoff criteria.

**Table 4.** McNemar’s Tests Comparing Bullying Perpetration Prevalence of the Definition-Based and the Behavior-Based Scale.

Comparison	Frequency	Grade	McNemar’s Chi-Squared	*p*	OR
DB vs. BB 1 item	2–3 Times/month	4	75.18	<.001	13.7
DB vs. BB 2 items	2–3 Times/month	4	19.32	<.001	4.3
DB vs. BB 1 item	Once a week	4	19.11	<.001	5.3
DB vs. BB 2 items	Once a week	4	0.45	.50	—
DB vs. BB 1 item	2–3 Times/month	5	98.22	<.001	15.3
DB vs. BB 2 items	2–3 Times/month	5	28.90	<.001	5.4
DB vs. BB 1 item	Once a week	5	30.95	<.001	7.1
DB vs. BB 2 items	Once a week	5	5.33	.021	2.9
DB vs. BB 1 item	2–3 Times/month	6	130.55	<.001	36
DB vs. BB 2 items	2–3 Times/month	6	36.82	<.001	9
DB vs. BB 1 item	Once a week	6	28.20	<.001	12.7
DB vs. BB 2 items	Once a week	6	3.37	.066	—
DB vs. BB 1 item	2–3 Times/month	7	181.99	<.001	21.2
DB vs. BB 2 items	2–3 Times/month	7	54.75	<.001	5.7
DB vs. BB 1 item	Once a week	7	84.49	<.001	13.5
DB vs. BB 2 items	Once a week	7	33.59	<.001	6.88
DB vs. BB 1 item	2–3 Times/month	8	172.18	<.001	14.3
DB vs. BB 2 items	2–3 Times/month	8	64.80	<.001	6.35
DB vs. BB 1 item	Once a week	8	72.78	<.001	7.86
DB vs. BB 2 items	Once a week	8	19.56	<.001	3.38

*Note*. OR = odds ratio; DB = definition-based scale; BB = behavior-based scale.

**Table 5.** McNemar’s Tests Comparing Bullying Victimization Prevalence of the Definition-Based and the Behavior-Based Scale.

Comparison	Frequency	Grade	McNemar’s Chi-Squared	*p*	OR
DB vs. BB 1 item	2–3 Times a month	4	422.33	<.001	38.2
DB vs. BB 2 items	2–3 Times/month	4	186.93	<.001	9.9
DB vs. BB 1 item	Once a week	4	205.23	<.001	17.6
DB vs. BB 2 items	Once a week	4	60.03	<.001	4.9
DB vs. BB 1 item	2–3 Times/month	5	406.87	<.001	49.8
DB vs. BB 2 items	2–3 Times/month	5	236.06	<.001	16.8
DB vs. BB 1 item	Once a week	5	275.41	<.001	58.4
DB vs. BB 2 items	Once a week	5	117.81	<.001	14.7
DB vs. BB 1 item	2–3 Times/month	6	408.58	<.001	34.5
DB vs. BB 2 items	2–3 Times/month	6	195.72	<.001	12.1
DB vs. BB 1 item	Once a week	6	193.23	<.001	20.5
DB vs. BB 2 items	Once a week	6	76.88	<.001	7.12
DB vs. BB 1 item	2–3 Times/month	7	530.77	<.001	56.2
DB vs. BB 2 items	2–3 Times/month	7	266.70	<.001	13
DB vs. BB 1 item	Once a week	7	302.29	<.001	33.3
DB vs. BB 2 items	Once a week	7	140.82	<.001	13.7
DB vs. BB 1 item	2–3 Times/month	8	437.48	<.001	65.7
DB vs. BB 2 items	2–3 Times/month	8	246.13	<.001	23.5
DB vs. BB 1 item	Once a week	8	269.42	<.001	57.2
DB vs. BB 2 items	Once a week	8	129.19	<.001	17.3

*Note*. DB = definition-based scale; BB = behavior-based scale.

## Discussion

Consistent with our expectations and previous research (Beltran-Catalan & Cruz-Catalan, 2020; Bjereld et al., 2020; Hellström et al., 2013; Huang & Cornell, 2015; Xie et al., 2023), the current findings showed that the percentages of bullies and victims were higher when the self-reported measure used a behavior-based approach compared to a definition-based approach. The unique contribution of this study is the examination of prevalence rates in a 4-year longitudinal design (five waves of data) where students were followed from grades 4 through 8 and answered both scales at each wave. Differences between the two measurement conditions were consistently significant across all waves when using 2 to 3 times a week as a cutoff value and irrespective of using either one or two items as the cutoff criterion for the behavior-based scale. When examining more frequent reports of bullying (i.e., once a week cutoff), there was one nonsignificant difference: the difference in the proportion of bullies was not significant in the fourth and sixth grades when comparing the definition-based measure to the cutoff of two items from behavior-based scale.

The behavior-based self-report bullying measure may have led to more honest reporting because it avoided the use of the word bullying which might have minimized the elicitation of stigma and negative emotions (Xie et al., 2023). In addition, both Vaillancourt et al.’s (2010) and Huang and Cornell’s (2015) findings suggest that specific behavior items on bullying may elicit greater recall from students than the global question on bullying. According to Xie et al. (2023), “the definition of bullying covers a wide range of behavior categories, but the sensitivity and cognition to different forms of bullying vary in participants. Therefore, listing a series of bullying behaviors and asking participants to choose the frequency of each behavior might be the best way to help them recall specific experiences and avoid imprecise reporting” (p. 252). Thus, behavior-based measures might also decrease the risk of perception and recall biases in comparison to definition-based measures.

In contrast, our findings could also be a result of behavior-based measures simply contributing to overreporting of these behaviors because they could be tapping into general types of aggression (see also Bjereld et al., 2020; Furlong et al., 2010). Indeed, measures that simply consist of a list of aggressive behaviors could be criticized for overreporting to bullying. However, our behavior-based stem did assess for power imbalance (e.g., bullied someone who is weaker, less popular, or less powerful in comparison to you/bullied by someone who was stronger), which is often not part of behavior-based measures (Martin et al., 2020). Volk et al. (2017) argue that a “power imbalance is perhaps the feature that most clearly differentiates bullying from other forms of aggression” (p. 35) and building on this criterion in behavior-based self-report bullying measures, like the one in the current study, would therefore counteract the risk of conflating bullying with aggression.

Higher prevalence rates of bullying involvement with the behavior-based measure could also be partially explained by the failure to assess intentionality directly. Instead, the items simply describe negative behaviors that usually are considered physical, verbal, or relational aggression that is unwanted and result in harm or discomfort on behalf of the target (e.g., “Pushed me so that it hurt, or so that I fell down,” “Teased me or called me mean names in a way that bothered me,” and “Spread mean rumors or lies about me”). Interestingly, the definition-based measure (Olweus, 1996a, 1996b) in this study also failed to assess the intention to inflict harm directly. More specifically, the definition stated that it would *not* be bullying “when the teasing is done in a friendly and playful way.” This part of the definition excludes other forms of bullying (physical and relational) and does not explicitly state that repeated negative behavior that produces harm or discomfort to someone in a weaker position is only bullying when there is intentionality on the part of the bully.

Historically, Olweus (1993) argued that intentionality to inflict harm was a critical feature of bullying that differentiated the behavior from accidental harm (Hellström et al., 2021). To make this distinction, he asked survey respondents to not include teasing that was friendly and playful teasing (Olweus, 1996a, 1996b), but again this falls short of assessing intentionality directly. In contrast to Olweus’ (1993) definition, both Gladden et al. (2014) and Volk et al. (2017) abandon this criterion in their definitions. For example, Gladden et al. (2014) state instead that “bullying is any unwanted behavior(s)” that “may inflict harm or distress on the targeting youth including physical, psychological, social or educational harm” (p. 7). In other words, Gladden et al. (2014) shift the focus from bullies’ intention of bullying to how it is perceived by and affects the victim. Intention to inflict harm is also particularly difficult to assess both on the part of children bullying others and those being bullied (Hellström et al., 2021). Asking students to estimate whether those who are bullying them had the intention to harm them on a bullying victimization scale would require them to guess or speculate about others’ motives, goals, and intentions. This would be highly vulnerable to perception bias.

In addition, asking students who are bullying others to estimate whether they had an intention to inflict harm assumes sufficient self-awareness and honesty. Further, research has shown that bullies and other peers may sometimes perceive bullying as playful teasing or a harmless and friendly joke (Betts & Spenser, 2017; Chandler, 2018; Forsberg, 2019; O’Brien, 2019; Teräsahjo & Salmivalli, 2003). Also, students who often bully others score higher than their peers in moral disengagement (Luo & Bussey, 2023; Thornberg, 2023). Students who bully are more inclined to hold social-cognitive distortions that their immoral conduct (e.g., bullying someone) is not morally wrong but acceptable or even desirable, and prevents any feelings of guilt, shame, and remorse (Bandura, 2016). Among the mechanisms of moral disengagement are *euphemistic labeling* (i.e., labeling the aggressive or harmful behavior in a way that makes it sound less negative or more respectable, such as talking and thinking about one’s bullying behavior as “just kidding,” “joking,” and “playful teasing”), and *distorting the consequences* (i.e., perceptually minimizing, ignoring, or misconstruing the harm or discomfort one’s behavior cause someone else). If these moral disengagement mechanisms are activated, the intention to inflict harm might be unnecessary or irrelevant as a motive for engaging in bullying perpetration (i.e., “I do not intend to do harm because my action is not mean, and no one gets hurt or harmed.”). Thus, asking students who bully others that are morally disengaged about intentionality will likely lead to underreporting.

### Limitations

This study is not without limitations. Although the within-subject design employed here was a particular strength of the study and an improvement to the extant literature, we did not control for possible order effects. All students completed the behavior-based measures before they completed the definition-based measures on the questionnaire. The decision to place the behavior-based measure before the definition-based measure was to avoid possible carryover effects of the bullying terminology, which would be a risk if the order was reversed (i.e., first letting the students respond to the definition-based measures and then the behavior-based measures). This decision was also supported by the findings of Huang and Cornell (2015), who found that when students responded to specific behavioral items on bullying victimization before answering a general question on bullying victimization, their prevalence rate in the later global measure increased. These authors argued that completing questions on specific types of bullying prior to the global question may lead to greater recall. However, future research should examine this further by randomizing the order across the participants.

Another limitation is that we used the original wording of both measures. More specifically, the definition-based scale asked the students to report their experiences of bullying “in the past couple of months” and the behavior-based scale asked the students to report their experiences of the specific behaviors in “the past three months.” The expression “couple of months” in the definition-based measure is less specific than the behavior-based measure and may have been interpreted as including 2 or 3 months. Thus, the behavior-based measure might be interpreted by the students as including 1 more month, which in turn might contribute to a higher prevalence. On the other hand, it is likely that the students found these two response options to be quite similar. Nevertheless, future research should standardize the response options across measures.

Finally, the sample was selected through a non-probability procedure of schools in Sweden. Although we conducted a strategic sampling procedure of schools to include a diverse sample regarding socioeconomic backgrounds and socio-geographic locations, we did not collect or control for data on socioeconomic background or ethnic group diversity at the individual level. Additionally, we did not gather data on or examine possible differences concerning other minority groups such as LGBTQ+ students and students with various disabilities. Future studies should replicate the current findings with other samples of students from different minority groups and cultural backgrounds, in which socioeconomic, ethnicity, and other possible background data are included in the analyses.

### Implications for Practice

The findings from the study have implications for school-based practice. Results highlight the need for transparency in measuring bullying. Considering that the effectiveness of bullying intervention and prevention programs has been modest in meta-analyses and research reviews (Gaffney et al., 2019; Jiménez-Barbero et al., 2016; Polanin et al., 2022), it is important to explore the extent to which efficacy varies depending on the type of bully assessment employed. Implementation of bullying assessments that yield reliable and valid scores is the first critical step in bullying prevention and intervention efforts. Further, it is imperative that these assessments consider that students’ definition of bullying can differ substantially from those definitions developed by researchers (Byrne et al., 2016). Practitioners and researchers alike need to deliberately consider pros and cons of using a behavior-based scale versus a definition-based scale. In the present study, the behavior-based scale yielded a higher prevalence of bullying perpetration and victimization than the definition-based scale across adolescence longitudinally when controlling for individual differences. It is possible that students can more accurately and honestly respond to experiences with bullying when they are provided with concrete and specific descriptions of behaviors rather than providing them with general, global, and potentially emotionally-laden bullying terminology. Even so, both approaches have their strengths and limitations, and practitioners must take into account the range of possible biases and the risk of underreporting and overreporting when choosing among the approaches to measuring school bullying. If they choose to include both measurement approaches in their student surveys, practitioners should place the behavior-based scale before the definition-based scale to avoid a negative carryover effect and to decrease the risk of underreporting in the definition-based scale due to recall bias (Huang & Cornell, 2015). Either way, it is critical to understand that bullying measures will never provide exact representations but only estimates or approximations of bullying prevalence.

## Conclusion

The current study, which compared behavior-based measures of bullying and definition-based measures of bullying, is a significant contribution to the assessment of bullying as “measurement is still the Achilles’ heel of bullying research” (Volk et al., 2017, p. 36). Effective bully prevention efforts rest on the ability to accurately assess the prevalence of bullying longitudinally with measures that resonate with youth and assess bullying apart from other forms of aggression. The behavior-based scales used in this study offer researchers and practitioners a self-report bullying measurement that includes power imbalance, concrete and specific negative behaviors, and the ability to estimate repetition, but without using bullying terminology. In this way, the behavior-based measure addresses the risk of underreporting due to recall bias as well as self-denial and social desirability biases that seem to plague definition-based scales in the larger extant literature. Accordingly, more students were identified as bullies and victims of school bullying in the current study when they completed the behavior-based scales as compared to the definition-based scales. Still, pros and cons of both approaches can be further discussed, and both definition-based and behavior-based self-report measures are vulnerable to a number of biases while they provide estimates or approximations—not exact pictures—of bullying prevalence.

## Supplemental Material

sj-docx-1-jiv-10.1177_08862605241262216 – Supplemental material for Prevalence Rates of Bullying: A Comparison Between a Definition-Based Scale and a Behavior-Based Scale Supplemental material, sj-docx-1-jiv-10.1177_08862605241262216 for Prevalence Rates of Bullying: A Comparison Between a Definition-Based Scale and a Behavior-Based Scale by Björn Sjögren, Ylva Bjereld, Robert Thornberg, Jun Sung Hong and Dorothy L. Espelage in Journal of Interpersonal Violence
