# Supplementary material for: Prevalence Rates of Bullying: A Comparison Between a Definition-Based Scale and a Behavior-Based Scale
Source: J Interpers Violence. 2024 Jul 31;40(7-8):1530–52. doi: 10.1177/08862605241262216 (PMC11874513; doi:10.1177/08862605241262216)
Supplement: sj-docx-1-jiv-10.1177_08862605241262216 – Supplemental material for Prevalence Rates of Bullying: A Comparison Between a Definition-Based Scale and a Behavior-Based Scale [file sj-docx-1-jiv-10.1177_08862605241262216.docx]

**Appendix A**

**The School Bullying Victimization Scale (SBVS)**

Think of the past three months. How frequently have one or more students who are stronger, more popular, or more powerful than you are, done the following things to you at school?

1. Joked around with me in a way that bothered me
2. Excluded me from their group
3. Pushed me so that it hurt, or so that I fell down
4. Teased me or called me mean names in a way that bothered me
5. Hit or kicked me to hurt me or make me feel bad
6. Left me out and didn’t let me hang out with them at recess
7. Broken my things
8. Spread mean rumors or lies about me
9. Held me against my will
10. Mocked or made fun of me in a way that bothered me
11. Taken things from me

The response options for each item: 1 = “Has not happened to me”, 2 = “Only a few times”, 3 = “2 or 3 times a month, 4 = “About once a week”, 5 = “Several times a week”

**The School Bullying Perpetration Scale (SBPS)**

Think of the past 3 months. How often have YOU done the following things at school to one or more students who are less strong, less popular or less powerful than you are?

1. Joked around with the student making him/her feel embarrassed
2. Excluded him/her from our group
3. Pushed the student so that it hurt, or so that he/she fell down
4. Teased the student and called him/her mean names
5. Hit or kicked the student to hurt him/her
6. Left the student out and didn’t let him/her hang out with us at recess
7. Broken his/her things
8. Spread mean rumors or lies about him/her
9. Held the student against his/her will
10. Mocked or made fun of the student in a way that bothered him/her
11. Taken things from him/her

The response options for each item: 1 = “I haven’t done this”, 2 = “Only a few times”, 3 = “2 or 3 times a month, 4 = “About once a week”, 5 = “Several times a week”
